# Methodologies of Autologous Skin Cell Spray Graft

**DOI:** 10.7759/cureus.31353

**Published:** 2022-11-11

**Authors:** Anand Shree, Anjali A Vagga

**Affiliations:** 1 Anatomy, Jawaharlal Nehru Medical College, Datta Meghe Institute of Medical Sciences, Wardha, IND; 2 Biochemistry, Jawaharlal Nehru Medical College, Datta Meghe Institute of Medical Sciences, Wardha, IND

**Keywords:** collagen, hydrogels, tissue engineering, spray graft, skin

## Abstract

Healthcare workers continue to struggle with skin wound management. Despite the fact that there have been many methods created throughout the decades for skin regeneration, contemporary developments in regenerative medicine provide highly reliable methods for creating synthetic skin replacements, such as electrospinning, 3D bioprinting, or spraying, among all others. Specifically, skin sprays have been a cutting-edge method that is currently undergoing clinical testing and has lots of promise for providing cells and hydrogels to cure both acute and chronic wounds. Deposition of cells and scaffolding materials in successive layers over the burnt regions characterises 3D bioprinting for burn injury repair. Depending on the desired outcome, bioprinting of skin may be performed in vivo or in vitro. However, the location of the printing and the time required for the tissue to mature separates these two methods. Bioprinted skin for use in burn repair faces technical and regulatory hurdles before it may be used in clinical settings. Skin sprays provide various benefits to traditional wound care methods, inclusive of ease of administration, the ability to treat broad wound regions, and a consistent dispersion of the sprayed substance. The most recent developments in this technology are reviewed in this article, along with a thorough explanation of investigational and presently marketed acellular and cellular skin spray solutions that are utilized to treat a range of illnesses and administer various experimental materials. Additionally, we describe regulatory processes for their capitalization and cover key clinical studies for various skin illnesses and associated treatment circumstances because skin spray products are susceptible to several classifications. Finally, we provide an argument and offer potential future developments in the biotechnology of skin sprays for improved clinical dermatological applications.

## Introduction and background

A collagen scaffold called artificial skin encourages skin regeneration in animals and humans. It has been shown that treating adult animals and people with scaffolds for serious skin wounds causes the dermis to regenerate [1].

The three layers of skin are the epidermis, dermis, and hypodermis. Every layer of the epidermis is made up of keratinized epithelium, which undergoes continual renewal. The primary epidermal cell type, keratinocytes, are essential for maintaining skin health because they may distinguish and create broad diversity of structural proteins and lipids necessary for epidermal regeneration [2]. This includes burns and various chemical and physical traumas, genetic disorders, skin issues (like diabetic foot ulcer, perianal fistulae, and autosomal recessive disease bullosa), and the elimination of skin during surgery. Skin lesions may be classed as epidermal, deep partial-thickness, superficial partial-thickness, or full-thickness based on the deepness of the lesion. Wounds must be recognized as soon as feasible for the best possible treatment, even if it is difficult to precisely identify certain instances. To cure epidermal and superficial partial-thickness wounds without the need for treatment, the skin relies on its self-healing powers [3].

**Figure 1:**
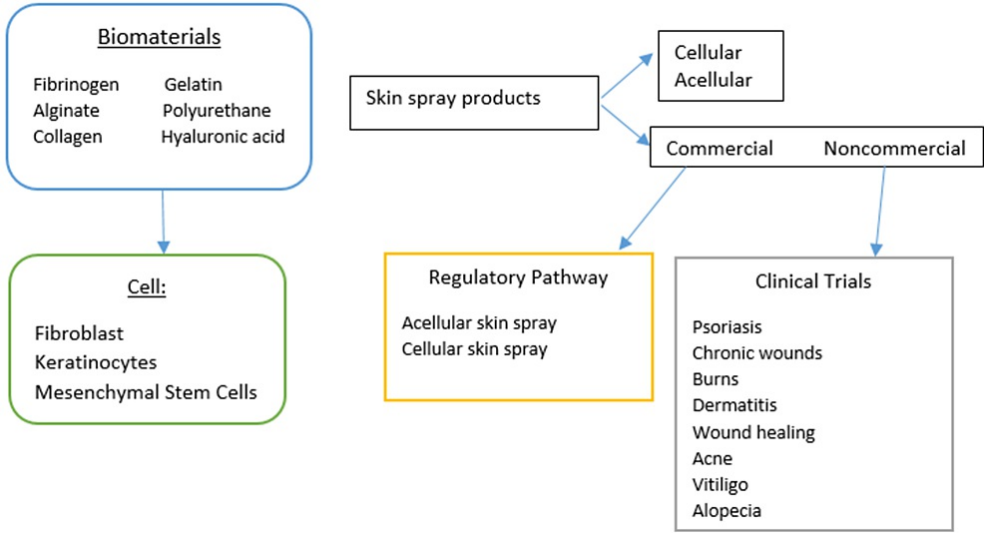
An overview of artificial skin

Figure 1 depicts an overview of artificial skin.

Dr Loannis V. Yannas and Dr John F. Burke developed a method for promoting skin regeneration. For decades, normal skin grafts have been utilized to repair severe wounds, despite the fact that they have some downsides and limitations of their own. Although early excision with autograft encapsulation is ideal, it may be challenging to perform in patients with significant burns because of the small size of donor sites [4]. While waiting for cultured epithelial autografts, a patient might acquire a systemic sickness or a wound infection locally, both of which would cause delays. In response to these limitations in auto graft coverage, researchers have developed a no-cultured epithelial copy that may be applied immediately after harvesting from tiny donor sites. Because of the initial challenges in delivering cultured epithelial autografts to wound beds, a spray-on approach for delivering proliferating keratinocytes has been developed. Tissue engineering and regenerative medicine have developed many treatment techniques for the rejuvenation of skin lesions with the use of different skin alternatives, such as cell suspensions, hydrogels, and 3D scaffolds. Although not widely used in clinical practice, skin sprays might be a potential choice for wound healing because of their large number of advantages, including their flexibility to carry different cell kinds and materials. Numerous synthetic and natural polymers were used in the creation of various kinds of grafts for testing and evaluation. Developed for commercial use under the trade name Integra, it treats chronic skin wounds, big burn victims, and skin plastic surgery [5]. Recent advancements in skin spray technology are discussed, including both in-progress and commercially available marketed acellular and cellular treatments, as well as a discussion of their regulatory processes to market, and the primary techniques for skin regeneration are summarised. Finally, we offer future directions for the bioengineering of skin sprays that will allow for more practical clinical use, and we include a thorough search for clinical studies employing sprays to treat a range of skin problems.

## Review

Skin graft

A sort of surgery is skin grafting. Medical practitioners perform this procedure to transfer healthy skin from one part of the body to another to cover damaged or missing skin. Within a matter of days, the transplanted skin forms blood vessels and then fuses with the skin’s surroundings. Skin transplant surgery is used to treat patients who have skin damage due to burns, wounds, diseases, or infections. The doctor may suggest a skin transplant to replace the skin that was removed during surgery to eradicate skin cancer [6]. Our general health, the amount of the transplant, and the approach the surgeon utilized will all influence how long it takes us to recuperate from this procedure. In most cases, skin transplant surgeries are successful on their first try. Split and full-thickness skin autografts, which entail removing a section of skin from a patient's body that hasn't been wounded and grafting it in place of the injured area, were two types of skin grafts used [7]. Third-degree burns are treated with this, but it hurts and requires more healing space. Autologous skin grafts, made from a sample of the patient's healthy skin and expanded through meshing procedures or cell culture, may be used to treat wounds. On the other hand, a patient's wound may be temporarily covered by skin grafts that were obtained from an animal or another person as a donor (allograft and xenograft, respectively) [8].

Three-dimensional bio-printing

It is an engineering and technology technique. By layering biomaterials and living cells over computer-aided designs, artificial skin tissues with specific designs for pores can be created. An ideal 3D bioprinting skin would be highly permeable, with a system of interlinked holes that would allow nutrients to be transported and wound exudates to be removed while also protecting the site from microbial invasion [9]. It would also be biocompatible and biodegradable, as well as having the requisite mechanical qualities, surface chemistry, and mechanical properties. While primary cells, stem cells, and heterologous cell lines are all viable options for bioprinting, the latter two are utilized far less often. Types of cells that fall into this category include keratinocytes, mesenchymal stem cells, fibroblasts, and induced pluripotent stem cells. Bone fragments, cells, chemical molecules, and extracellular matrices are just a few examples of the kinds of biological materials that may be printed with the help of 3D bioprinters. Its usefulness in analysing drug effects has recently been shown [10]. The success rate of surgeries using printed tissues and organs has increased in recent years. In order to prevent the body from rejecting donor tissue, scientists have employed 3D bioprinting to construct a layer resembling skin with a blood artery. It has been established in vitro that dermal fibroblasts release soluble substances that may affect keratinocytes to stimulate the development of bottom membrane proteins or melanogenic factors [11]. Keratinocyte growth and the formation of distinct keratinocyte layers are both encouraged by dermal fibroblasts. Therefore, in basic 2D feeder-layer co-cultures, the coupling of post-mitotic fibroblast cells (federal cells) with epidermal keratinocytes does not result in adequately stratified epithelia [12].

Skin spray

Topical sprays have become increasingly popular in medical practice over the past couple of decades as a process for applying hydrogels or cell suspensions to cure both severe and long-term lesions because of their advantages, including the capability of treating large injuries over topographically challenging areas, the relatively short application time, and the homogeneous division of the sprayed suspensions [13]. Paying close observation to the sprinkling limits, such as the spraying pressure, the distance between the spray device's top and receiving surface, the angle, and the sprayed volume, is crucial when utilizing a spray device to transport nanomaterials and live cells for medical purposes. For instance, it has been shown in clinical studies that a minimum distance of 10 cm must be maintained between the spray device and the body for a secure delivery that does not increase the risk of an air embolus. The concentration of the conveying fluid, nozzle diameter, range, droplet velocity, and the characteristics of the receiving surface are just a few of the variables that can affect whether or not sprayed cells survive when they come into proximity to the target area [14]. It is also anticipated that sprayed cells may sustain damage when they come into proximity to the specifically aimed area. It has been shown that the post-aerosolized cell viability may be greatly decreased by increasing the pressure, decreasing the nozzle diameter, increasing the transport fluid density, increasing the spraying velocity, and increasing the flexibility of the recipient tissue surface. On the contrary, a larger droplet width that contains cells has a positive influence on cell viability. However, concentrating just on the survivability after aerosolization is inadequate. Spraying cells exposes them to three forms of stress (hydrostatic, tensile, and ultimate tensile), all of which have the potential to injure cells without breaking their membranes, necessitating an assessment of their proliferative ability. The amount of time under stress has a significant impact on the severity of the resulting impairment [15].

Discussion

Biomaterials Used in Skin Substitutes

Natural and synthetic biomaterials have all been utilized extensively as skin replacements. Three-dimensional polymeric networks, similar to those found on the surface of the skin, are created as a platform for the identification, adherence, and differentiation of cells in the body. The physiologic characteristics of the skin may be replicated by using natural materials, including fibrinogen, collagen, gelatin, hyaluronic acid (HA), and silk. This has been accomplished [16]. Collagenhas remarkable biocompatibility and is extremely biomimetic, which is due to the fact that it is the principal structural protein of the dermal extracellular matrix (ECM). Additionally, it has a porous surface that is good at permeability, low in immunogenicity, and has great biodegradability. Dressings made of collagen may be purchased in a number of forms, such as sheets, gels, lattices, and sponges, among other configurations. However, due to the prohibitive cost of using pure collagen, the biostability and mechanical strength of these dressings are sometimes lacking. Additionally, wound contraction and fibrosis may be noticed in each and every one of these dressings. It is possible to boost collagen's mechanical qualities by cross-linking it either physically or chemically or by mixing it with other substances such as glycosaminoglycans (GAGs), agarose, or chitosan [17]. A significant amount of fibrinogen, an important biopolymer in the process of wound healing, has been produced through the use of fibrin glues or sealants, which are used to close the wound and provide an instantaneous temporary composite that can be penetrated by cells that are working to repair the wound bed [18]. Thrombin is the component of the clotting cascade that is accountable for the transformation of fibrinogen into a fibrin clot. Because fibrinogen is biocompatible and biodegradable, it may be taken from the patient's own blood, which helps to reduce the risk of the patient developing an immune response to the substance [19]. It has also been shown that fibrin hydrogels may reduce the amount of scarring that occurs while simultaneously fostering increased wound healing, epidermal regeneration, and vascular expansion. One of the reinforced polymers with the poorest mechanical qualities is fibrin, which is also one of the most pliable. However, in addition to this, it has an elastic modulus deformation capacity and a large stretchability. On the other hand, fibrin gels have the potential to be improved by modifying variables such as pH, the quantity of fibrinogen, and thrombin, or by mixing fibrin with other chemicals such as HA and gelatin or polyethene glycol (PEG) [20].

Chitosan is a biomaterial generated from de-acetylated chitin that has been found to help in tissue regeneration and the healing of wounds. Some of the benefits of chitosan are that it is biocompatible, biodegradable, hemostatic, and antibacterial, and it stimulates the re-epithelization of wounds [21]. It is also possible to rapidly and easily transform it into a film, gel, or sponge. However, it has to have higher mechanical characteristics since pure chitosan cannot be utilized to manufacture skin replacements because it suffers from excessive shrinkage and distortion after drying. Chitosan needs to have improved mechanical qualities in order to be used to create skin substitutes [22]. Chitosan is often cross-linked, derivatized by structural change, or combined with other synthetic and natural polymers, such as gelatin, agarose, or collagen, in order to circumvent these limits. Other methods include chemically modifying chitosan. For instance, Soriano-Ruiz et al. (2019) developed chitosan hydrogels for use in the process of wound healing by using quaternary mixtures of three different glycosaminoglycans (HA, chondroitin sulphate, and dermatan sulphate) [23]. Gelatin, a collagen-derived protein, has been investigated as a sponge or film. It facilitates the formation of granulation tissue, has excellent biodegradability and biocompatibility, and is less costly than collagen. Due to its low mechanical strength, however, it can only be utilized efficiently when mixed with other polymers. Hydrogels composed of gelatin and chitosan, for instance, may be fortified by including HA or polycaprolactone to increase their mechanical strength. The mechanical strength of hydrogels increases when this combination is made.

Silkworm fibroin is excellent for healing wounds. It is non-carcinogenic and non-toxic, has high biological and ecological, hemostatic properties, possesses minimal immunogenicity, and is non-inflammatory [24]. In addition, it is considered to increase wound healing, promote cell growth, collagen formation, and re-epithelialization, and facilitate cell attachment. Silk fibroin is an interesting biomaterial for skin tissue engineering owing to its excellent mechanical characteristics and ease of manufacture. However, because of the absence of antibacterial properties in silk fibroin, wound infections are common. This problem may be overcome, though, by including more antibacterial substances [25]. Silk fibroin is often used in films, hydrogels, sponges, and nanofibrous scaffolds for skin wound healing applications, either in isolation or in combination with alginate or chitosan, among others. In a study conducted by Roh et al. (2006), utilizing a rat full-thickness wound model, the researchers investigated the effects of three sponge types on wound healing: a silk fibroin sponge, an alginate sponge, and a composite silk fibroin/alginate sponge [26]. They observed that all three sponge types increase wound healing relative to the standard, but the blended sponge had the greatest effect [27].

Biocompatibility and biodegradability, low cost, low toxicity, non-immunogenic properties, and the ability to chelate have made alginate, a naturally occurring polysaccharide polymer generated from seaweed, popular in the recent decade. Because of its hemostatic and moderate antibacterial characteristics, conformability, stimulation of granulation tissue growth and rapid epithelialization of wounds, alginate is used in a range of wound dressings, such as hydrogels, electrospinning mats, and sponges, to promote wound healing [28]. Due to alginate's strong hydrophilicity and capacity to hold water, a moist wound environment may be maintained without the risk of the wound bed drying up. This makes it a crucial material for wound treatment since it prevents additional harm when alginate-based dressings are torn off. There is no signal sequence in its structure; thus it cannot adhere to cells. Mixing alginate with other compounds (such as chitosan, fibrinogen, or gelatin), immobilising particular ligands, or cross-linking it might enhance its mechanical and structural integrity [29]. The most popular method of gelling alginate involves ionic cross-linking utilising divalent cations like Ca2+, despite the fact that these divalent cations may be released and exchanged with monovalent cations within the external medium, ultimately leading to the gel's dissolution. Alginate hydrogels may be gelled using a variety of methods, including covalent cross-linking, thermal gel formation, cell cross-linking, and free radical polymerization [30].

Because of their crucial functions in all stages of wound healing, glycosaminoglycans (such as HA, chondroitin sulphate, heparin sulphate, dermatan sulphate, and keratan sulphate) are crucial components in formulations for epidermal regeneration [31]. Besides its excellent biocompatibility and biodegradability, HA also has beneficial anti-inflammatory, mucoadhesive, and viscoelastic properties. Another benefit is that it helps keep tissues from sticking together or forming scar tissue. Although several HA-based products are now on the market, soluble HA's limited mechanical properties and rapid hyaluronidase degradation limit its therapeutic utility [32].

Synthetic polymers, on the other hand, may be tailored to give a wide range of physical qualities and are less costly and more dependable sources of material. Among these, polyurethane (PU) is preferred for wound healing because of its biocompatibility, high mechanical qualities, and ability to maintain a moist environment despite being permeable to gas but not water and germs [33]. Although PU materials are inexpensive, they only somewhat attach to the wound bed. Mesh made of nylon, poly(lactic acid), and poly(glycolic acid) have been utilized commercially as skin replacements. However, due to their poor rates of cell attachment and proliferation as a result of the restricted biological signals, alternative polymers, such as polycaprolactone or poly(L-lactide), have demonstrated minimal clinical effectiveness [34].

**Figure 2:**
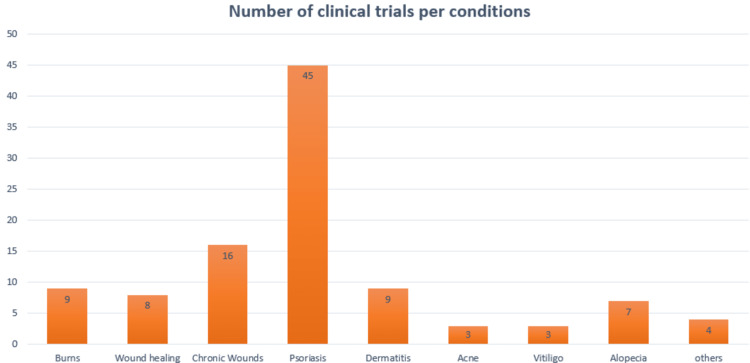
Number of clinical trials per condition

Despite their low mechanical strength, tendency to shrink or contract, difficulty in handling, and sometimes high costs, natural polymers often exhibit good biocompatibility and facilitate cell adhesion and proliferation [35]. The biological signals contained in natural polymers are absent from synthetic polymers, which, on the other hand, exhibit exceptional mechanical properties. A variety of natural and synthetic polymer combinations are frequently used to improve mechanical properties while still maintaining good biocompatibility, including cross-linking hydrophilic polymers, coating synthetic meshes with natural polymers, and creating hybrid meshes by embedding microspheres of natural polymer into synthetic knitted mesh openings [36]. Clinical skin replacements come in a variety of forms, including hydrogels, films, cell suspensions, cell sheets, and 3D skin scaffolds. The various biomaterials previously mentioned are used to create these products, as well as, in the case of cellular replacements, either autologous or allogeneic cells. These cells can be used right away, or they can first be grown to increase their population before being used to make skin replacements [37]. Figure 2 shows the number of trials per skin condition.

## Conclusions

A significant number of medical professionals continue to struggle with skin regeneration, particularly in cases of serious wounds. Even if there are a lot of various ways to tackle the problem, none of them provides an ideal solution. Sprays may be used as a delivery vehicle for hydrogels and cells, making it possible to treat vast damaged regions with a lot more ease and speed than with other methods, such as conventional skin sheet grafts or bioengineered skin replacements. There are currently many other types of skin sprays available; nevertheless, cell grafting is the procedure that has received the most attention in clinical studies. Fibrin, which is sometimes used as a hydrogel by itself and other times as a cell carrier, is the material most often used in experimental and commercially accessible skin spray therapies. Typically, epidermal cells such as keratinocytes and fibroblasts are used in cellular sprays for skin regeneration; however, the addition of stem cells or growth factors may enhance the stimulation of skin healing. Rheological investigations must be performed at various stages of production in order to optimise the mechanical features of the hydrogels used in skin sprays. Additionally, the viscosity of hydrogels employed in cellular sprays may impact the viability of cells. The ideal hydrogel should have shear-thinning features that reduce its viscosity while passing through a nozzle while maintaining robust mechanical properties to minimize cellular injury. There are viscoelastic characteristics synthesized since the skin-applied materials must be able to withstand body motions. There are a lot of acellular spray therapies that use drugs like corticosteroids to alleviate irritation brought on by conditions like psoriasis, but relatively few scientific trials investigating cellular regeneration spray remedies. Because of this, it is imperative to produce more therapeutically appropriate skin regeneration sprays that include hydrogels and cells. There are millions of people worldwide who suffer from skin conditions such as scarring or wound infection who might benefit from this treatment.
